# Molecular genetic study of novel biomarkers for early diagnosis of oral squamous cell carcinoma

**DOI:** 10.4317/medoral.20229

**Published:** 2014-12-05

**Authors:** Kim Yong-Deok, Jeon Eun-Hyoung, Kim Yeon-Sun, Pang Kang-Mi, Lee Jin-Yong, Cho Sung-Hwan, Kim Tae-Yun, Park Tae-Sung, Kim Soung-Min, Kim Myung-Jin, Lee Jong-Ho

**Affiliations:** 1 DDS, PhD. ProfessorDepartment of Oral and Maxillofacial Surgery, Graduate School of Dentistry, Seoul National University, Seoul, Republic of Korea; 2 Diagnostic DNA chip center, Institute of Medical Research, Seoul National University, Seoul, Republic of Korea; 3 Department of Oral and Maxillofacial Surgery, Ajou University School of Medicine, Suwon, Republic of Korea; 4 Department of Oral and Maxillofacial Surgery, Korea University Guro Hospital, Seoul, Republic of Korea; 5 Department of Statistics, College of Natural Sciences, Seoul National University, Seoul, Republic of Korea

## Abstract

Objectives: Early detection and treatment of an oral squamous cell carcinoma (OSCC) is critical because of its rapid growth, frequent lymph-node metastasis, and poor prognosis. However, no clinically-valuable methods of early diagnosis exist, and genetic analysis of OSCCs has yielded no biomarkers. Study Design: We investigated the expression of genes associated with inflammation in OSCCs via a quantitative reverse transcriptase polymerase chain reaction (qRT-PCR) analysis of microarray data. Tumor and normal tissues from five patients with an OSCC were used for microarray analysis. Differentially-expressed genes, identified using permutation, local pooled error (LPE), t-tests, and significance analysis of microarrays (SAM), were selected as candidate genetic markers. Results: Two groups corresponding to tissue identity were evident, implying that their differentially-expressed genes represented biological differences between tissues. Fifteen genes were identified using the Student’s paired t-test (p<0.05) and the SAM, with a false discovery rate of less than 0.02. Based on gene expression, these 15 genes can be used to classify an OSCC. A genetic analysis of functional networks and ontologies, validated by using a qRT-PCR analysis of the tissue samples, identified four genes, ADAM15, CDC7, IL12RB2 and TNFRSF8, that demonstrated excellent concordance with the microarray data. Conclusions: Our study demonstrated that four genes (ADAM15, CDC7, IL12RB2 and TNFRSF8) had potential as novel biomarkers for the diagnosis and the treatment of an OSCC.

** Key words:**Biomarker, microarray, quantitative reverse transcription polymerase chain reaction, oral squamous cell carcinoma, gene expression profiling.

## Introduction

An oral squamous cell carcinoma (OSCC), a subtype of head and neck squamous cell carcinomas, is the sixth most common malignancy worldwide, accounting for 3% of all cancers (1,2), and it remains one of the most intractable malignancies due to its invasive growth pattern, frequent cervical lymph-node metastasis and high recurrence rate (3). Approximately two-thirds of patients with an OSCC exhibit an advanced stage (Stage III or IV) at diagnosis because of its similarity to inflammatory disease, its long asymptomatic period and its challenging clinical differentiation (4,5). Therefore, early detection of an OSCC is critical. For that purpose, screening methods, such as light-based screening or brush cytology, have been introduced, but their sensitivity and specificity are insufficient compared with those of scalpel biopsy. Hence, histopathological examination is still a cornerstone of diagnosis; however, excluding the possibility of misdiagnosis completely is difficult due to the inherent vagueness of the histopathological characteristics of an OSCC and variations in the pathologists’ experience.

Recently, mRNA biomarkers for an OSCC in serum or tissue have become new diagnostic and therapeutic targets because suitable biomarkers can improve the power, availability and cost-effectiveness of high-throughput screening for genetic alterations. Microarray analysis, validated by using the quantitative reverse transcriptase polymerase chain reaction (qRT-PCR), has been used to identify the genes underlying OSCC pathogenesis, which include IL-8 and VEGF (6). Some investigators have employed a multiple-gene model of 25 biomarkers with 86%-89% accuracy (7). However, no studies have established clinic ally-valuable biomarkers, which may be due to a lack of studies with tumor-normal (TN) paired matching of patients (8) or to the limited predictive power of micro array-based models to correlate the clinical endpoint with gene expression (9). In this study, we investigated the expression of genes associated with cancer and inflammation in patients with an OSCC by using microarray analysis, which was validated by using the qRT-PCR. These results were assessed by bioinformatics verification. With this approach, we tried to identify novel biomarkers for OSCC.

## Material and Methods

1.1. Patients

TN-paired tissues from five OSCC patients were used for the microarray analysis. Subsequently, tissues from 17 OSCC patients (TN-paired) were used for the qRT-PCR analysis. Fresh tissue samples were collected after obtaining written informed consent from 34 consecutive patients undergoing therapeutic surgical resection for an OSCC between 1 June 2011 and 28 June 2012. The patients’ characteristics are outlined in  Table 1. After excision, tissues were preserved immediately in RNAlater® solution (Invitrogen, USA) until use and were transferred to the laboratory on ice. This trial was approved by the Institutional Review Board at Seoul National University Dental Hospital and was conducted in full accordance with the Declaration of Helsinki.

**Table 1:**
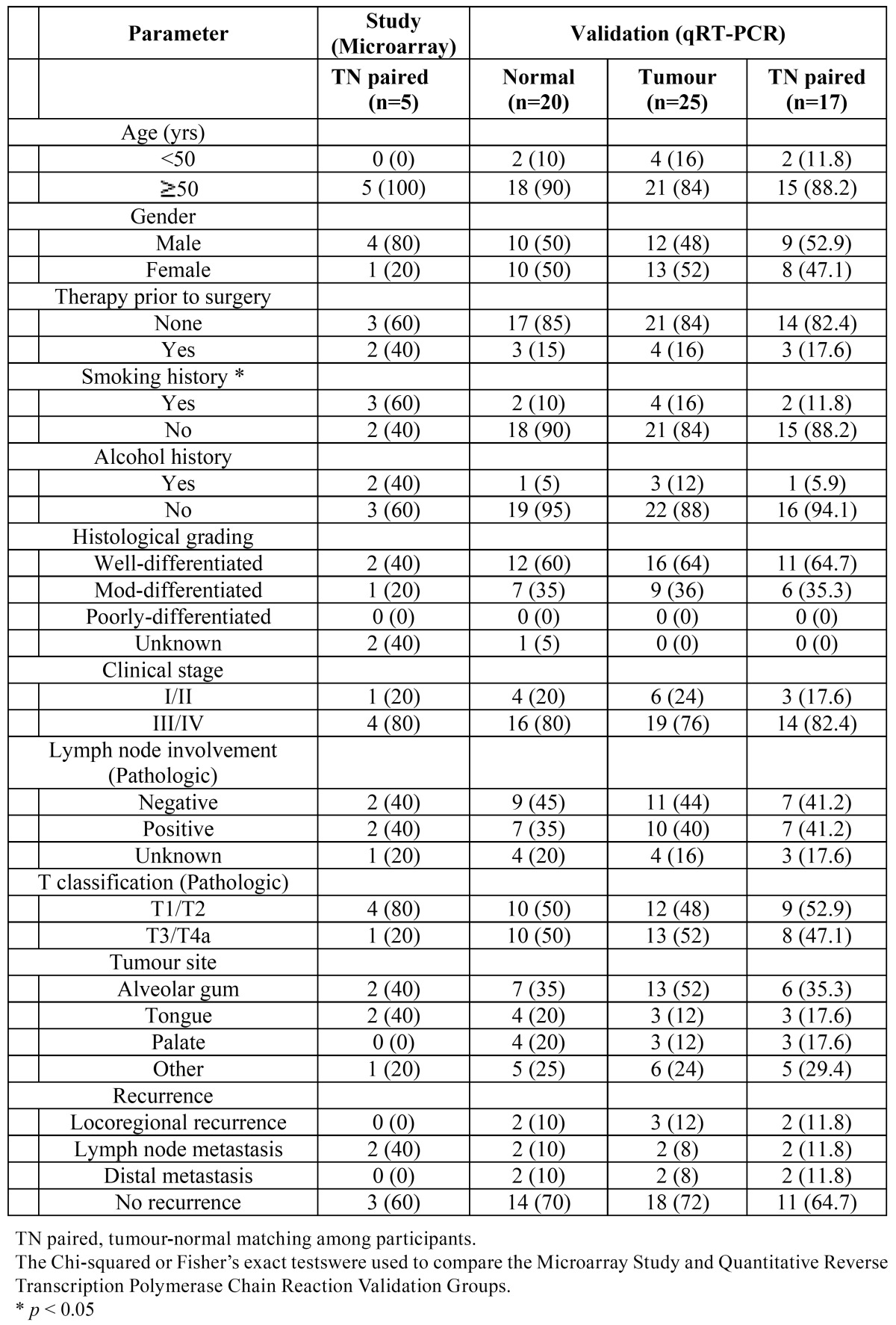
Clinicopathological characteristics of patients in this study and the Qualitative Reverse Transcription Polymerase Chain Reaction Validation Group.

1.2. RNA extraction and cDNA synthesis

RNAlater® was pipetted off the pellet, and the pellet was then washed with ice-cold phosphate-buffered saline, which was removed after centrifugation. Total RNA was extracted from the tissue samples by using the RNeasy® Mini Kit (Qiagen, Germany) according to the manufacturer’s instructions. To quantify and analyze the integrity of RNA, we used 2100 Bioanalyzer® (Agilent Technologies, USA). We reversely transcribed 10 μg of total RNA in the presence of an oligo (dT) T7 primer (iScript™ Select cDNA Synthesis Kit; Bio-Rad Laboratories, USA). cDNA was used for in-vitro transcription amplification in the presence of biotinylated nucleotides.

1.3. Microarray analysis

Gene expression was analyzed using the Affymetrix preparation protocol before hybridization to a GeneChip® 1.1 human genome microarray (Affymetrix, USA). Quality control of the arrays was achieved through analysis of the 5’:3’ ratios (range: 0.40-0.79), the percentage present (range: 37-47%), the average pair-wise correlation, and the principal component. Affymetrix® Microarray Suite version 5.0 (Affymetrix) was used for image processing and data acquisition. Gene expression levels and individual exon signal estimates contained in*. CEL files within the GeneChip® 1.1 platform (Affymetrix) were derived by using the robust multi-array average (RMA) algorithm, as implemented by the Expression Console v1.1.1 (Affymetrix). The quality of the data was assessed by analyses of the mean overall expression and its standard deviation, the detectable probe ratios, the control probe’s (housekeeping genes) expression data, and the correlation between samples from the same tissue.

1.4. Quantitative reverse transcription polymerase chain reaction

Target and housekeeping gene sequences were retrieved from the GenBank® database (National Center for Biotechnology Information (NCBI), USA), and applied primers were manually designed using the Primer-BLAST® tool (NCBI). Primer sequences are listed in  Table 2. RNA mixtures were subjected to first-strand cDNA synthesis using forward primers. The endogenous housekeeping genes, hypoxanthine guanine phosphoribosyl transferase (NM_000194.2) and β–actin (NM_001101.3), were used for data normalization, and relative quantification was performed by using a relative standard curve analysis with a CFX Connect™ Real-Time PCR Detection System (Bio-Rad Laboratories) and SYBR® Green I (Bio-Rad Laboratories) detection. cDNA (10ng) was dissolved in iQ™ SYBR® Green Supermix. The PCR comprised an initialization cycle (95°C for 3 minutes); 39 cycles of 95°C for 10 seconds, 56°C for 10 seconds, 72°C for 20 seconds, and plate reading at 80°C; and a final melting step at temperatures ranging from 56 to 95°C at a heating rate of 0.5°C/10 seconds, which was performed to create melt curves. The qRT-PCR was performed in quadruplicate for each cDNA samples, and negative controls with no template were included for each primer pair. Threshold cycle (Ct) values and target gene expression levels were calculated using CFX Manager™ Software (Bio-Rad Laboratories). The fold change of target gene expression in each treated sample, relative to the control sample, was derived from the negative Ct value: Ct (GAPDH)−Ct (target gene).

**Table 2:**
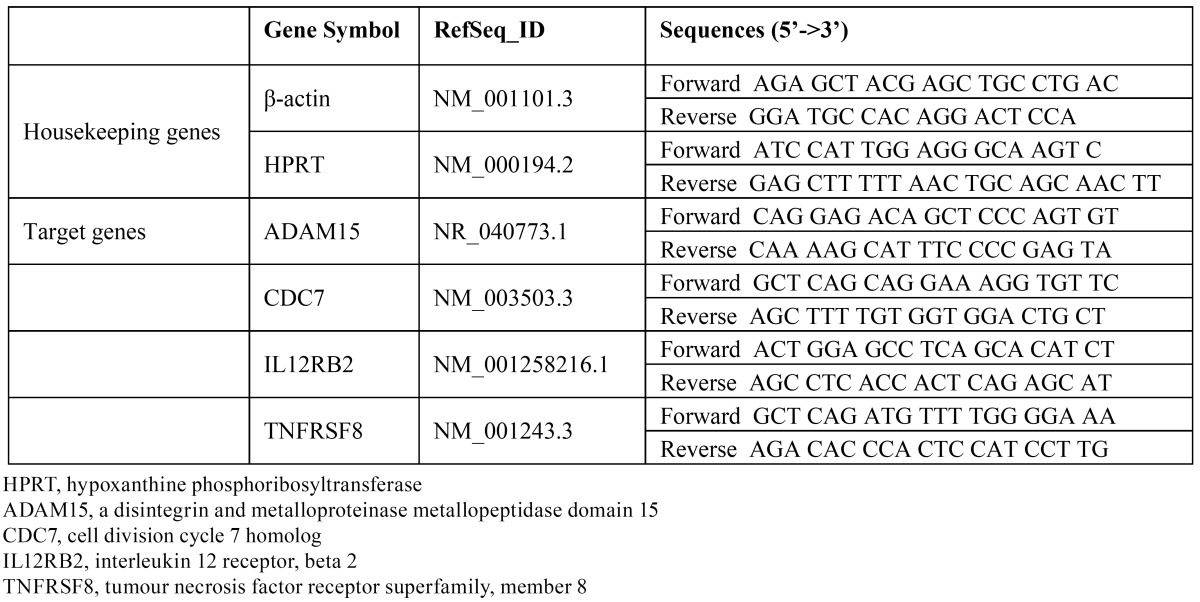
Primer pairs for reverse transcription polymerase chain reaction validation of candidate biomarker gene expression levels.

1.5. Data analysis and statistics

Differences in the clinical characteristics between the Microarray Study and the qRT-PCR Validation groups were compared using the Chi-square test. If this method was not suitable, a Fisher’s exact test or a linear-by-linear association test was used. Differentially expressed genes (DEGs), identified by using four statistical tests (permutation test, local pooled error (LPE) (10), Student’s paired t-test, and significance analysis of microarrays (SAM) (11), were selected as candidates for genetic biomarkers (12), and their normalities were tested using the Shapiro-Wilk test (13). After the normality had been tested, genes that were normally distributed were assessed using the t-test, the SAM and the LPE (parametric methods). The genes that were not distributed normally were assessed using a non-parametric permutation test.

For the multiple-comparison problem, p-values were adjusted using the false discovery rate (FDR) correction. To find candidates, we tried to use FDR values as close to 0% as possible, and those for the SAM and the permutation test were set to 0%. On the other hand, the FDRs for the LPE and the t-test were set to 0.001% and 0.02%, respectively, because no significant candidate genes were found when their FDRs were set to 0%. A discriminant analysis was used to find correlations between the candidate gene expressions and clinical parameters such as tissue heterogeneity, site specificity, and stage differences. From receiver-operating-characteristic (ROC) curves, the area under the curve (AUC), the sensitivity, the specificity, and the predictive value of each candidate gene were calculated using the Youden index. All statistical analyses, except the analyses of the ROC curves, which were done using MedCalc® Statistical Software version 12.7.8 (MedCalc® Software bvba, Ostend, Belgium), were conducted using R Statistical Software version 2.15.0 (from http://www.r-project.org/) and IBM SPSS® version 20 (IBM Co., Armonk, NY, USA).

## Results

2.6. Patient characteristics

Thirty-three patients with an OSCC, 17 men (51.5%) and 16 women (48.5%), were included in this study ( Table 1). We analyzed the DEGs in five patients by using microarray analyses to compare primary TN-paired tissue samples. Tumor (n=20) and normal tissues (n=25) from another 28 patients were used for the qRT-PCR validation. Of those 28 participants, 17 were assayed for expression via the qRT-PCR of primary tumor samples and matched normal mucosa. No statistically significant differences in clinical characteristics between the Microarray Study and the qRT-PCR Validation groups were observed (*p* > 0.05).

2.7. Statistical analysis of candidate genes identified by using a microarray analysis 

**Figure 1:**
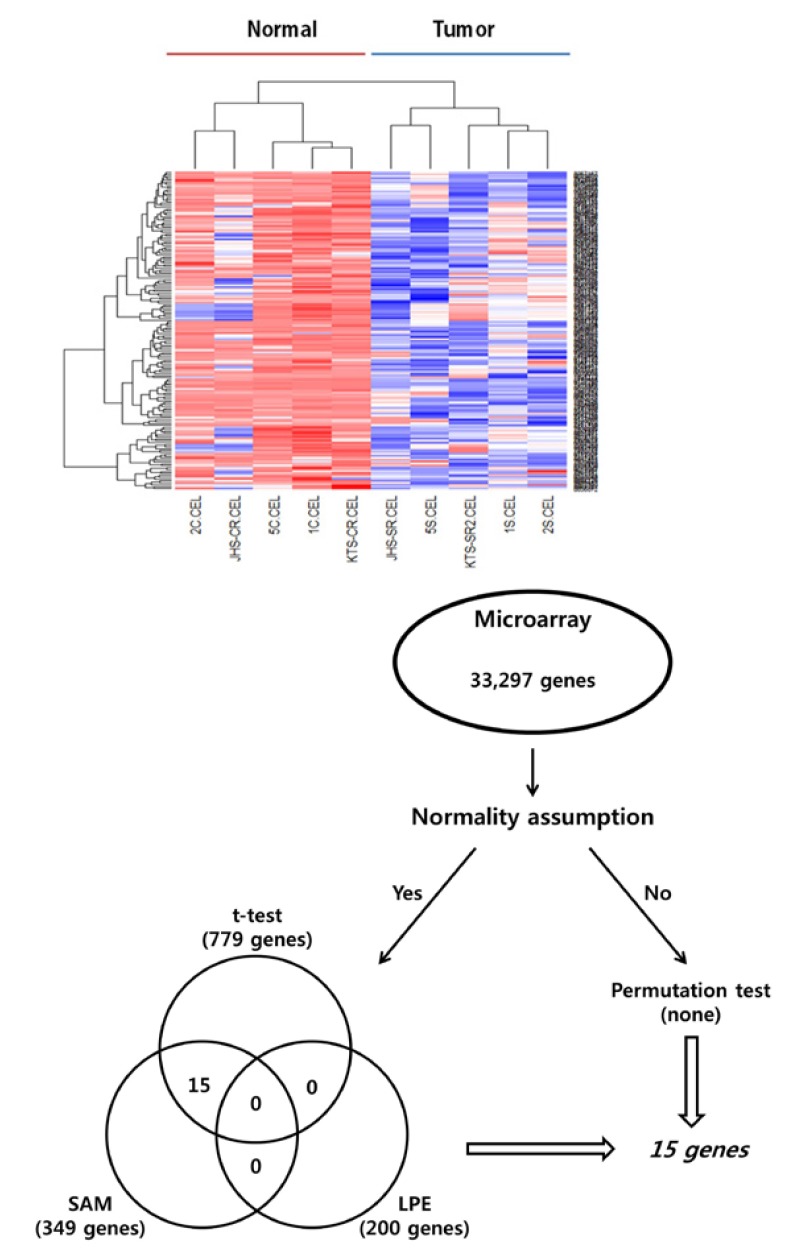
Hierarchical clustering of microarray gene expression data. The dendogram at the bottom lists all samples arrayed and measures their degree of relatedness in terms of gene expression. All samples were coded with numbers, as shown in table 1. The colored bar beneath the sample identifiers marks samples from patients, where the Normal Group of patients with oral squamous cell carcinoma are in red and those from the Tumour Group are in blue (A). Genes identified by all four statistical tests (the permutation, LPE and t-tests and SAM) were selected as candidate genetic markers. The 15 genes passed Student’s paired t-test and SAM analysis (B).

A t-test was performed to detect the DEGs. However, the test assumes that all data follow a normal distribution. Therefore, a non-parametric permutation test was also conducted to identify DEGs, and its results were evaluated using the SAM and the LPE test. The LPE test was proposed to overcome the limitation caused by the different error variances arising from the diverse biological conditions via local error estimation within quartiles and non-parametric smoothing (10). The SAM scores gene expression relative to the deviation of repeated measurements in order to identify genes with high scores above an adjustable threshold, and for those identified genes, the FDRs and the percentage of identification by chance were estimated (Fig. 1) (11).

Of the 33,297 genes on the microarray, the numbers of genes with significance, which were assessed with the t-test, the SAM and the LPE (parametric methods), were 779, 349 and 200, respectively. None of these genes showed statistical significance in all 3 methods, but 15 genes did in both the t-test and the SAM. However, no genes showed significance in the non-parametric permutation test. The 15 genes showed differential expression patterns; 10 genes were predominantly upregulated and five were predominantly downregulated ( Table 3). Four statistical tests (the permutation test, the LPE, the Student’s paired t-test, and the SAM) were used to identify the DEGs, and common DEGs identified by using the t-test and the SAM were selected as biomarker candidates.

**Table 3:**
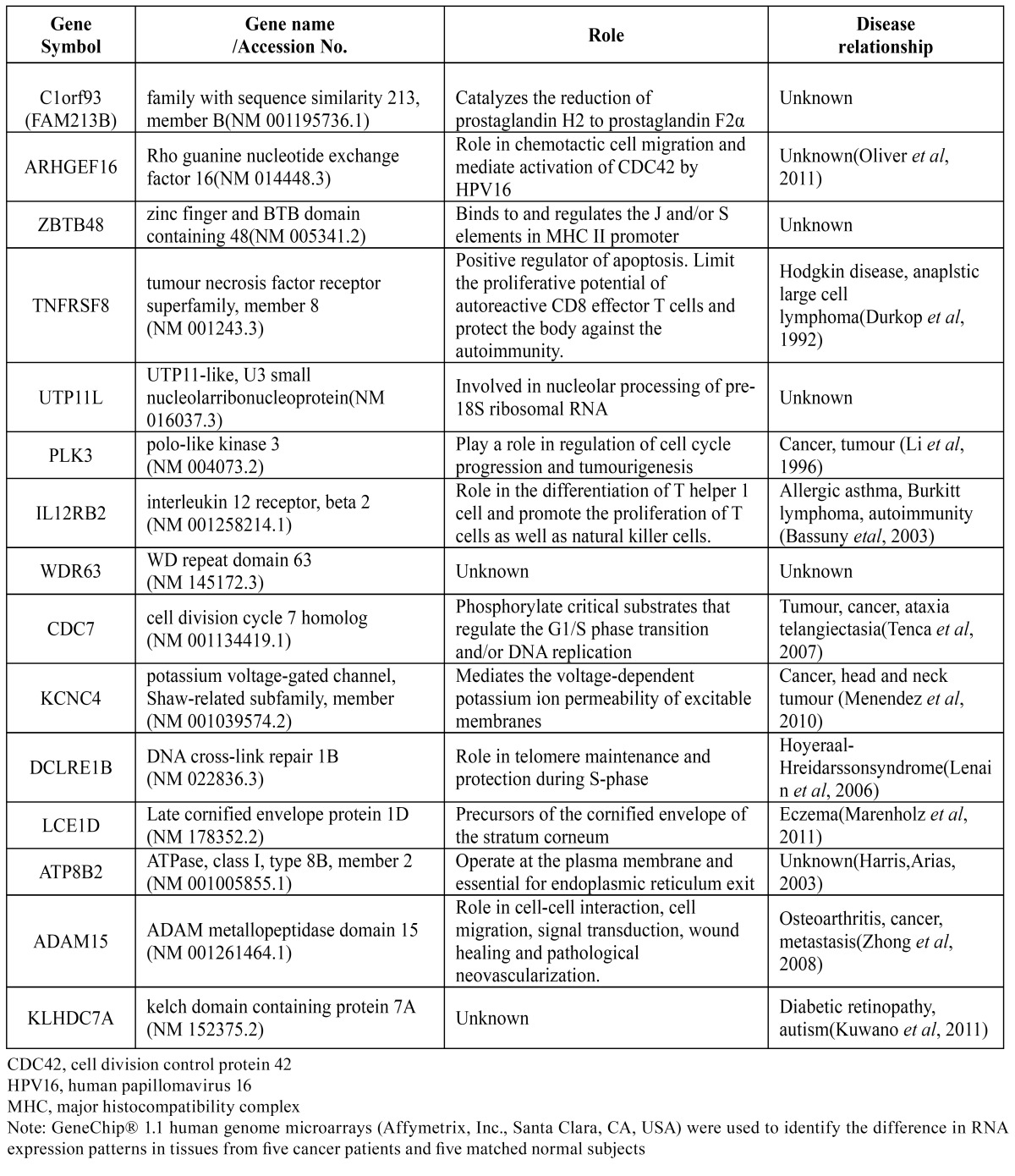
Fifteen candidate genes whose expression differed between normal and tumour tissue in oral squamous cell carcinoma as indicated by microarray analysis (tumour–normal-paired; n = 5).

**Figure 2:**
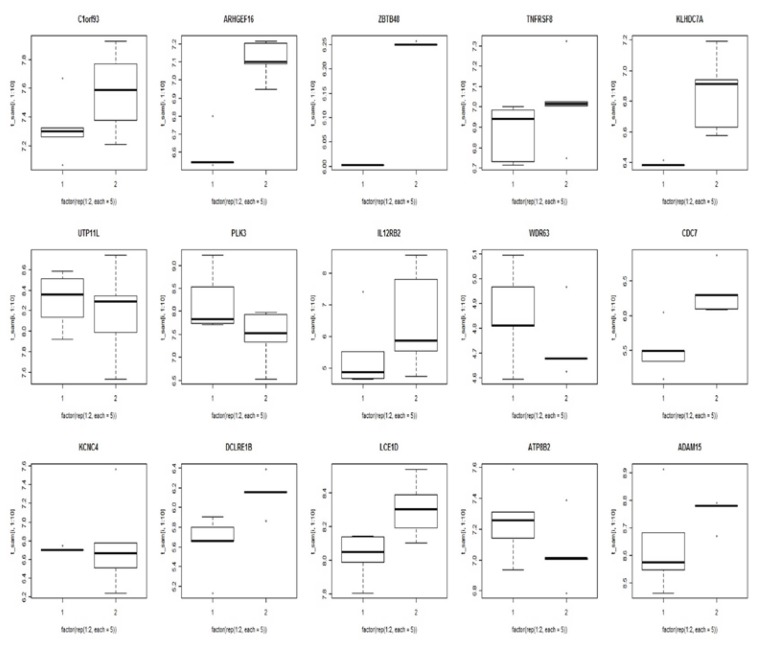
Differential expression of 15 genes selected by microarray in oral squamous cell carcinoma and normal tissue. Five genes were downregulated, while the other 10 were upregulated. The full line corresponds to the median value for each group.

Fifteen genes passed the Student’s paired t-test (*p* < 0.05) and the SAM analysis with an FDR of <0.02. To test the ability of this signature gene set to classify the OSCC and the Normal groups, we performed an average linkage hierarchical clustering analysis. Based on gene expression, this 15-gene set has classification power for an OSCC (Fig. 2). In this set, upregulated genes were FAM213B, ARHGEF16, ZBTB48, DCLRE1B, LCE1D, KLHDC7A, TNFRSF8, IL12RB2, CDC7 and ADAM15 while UTP11L, PLK3, WDR63, KCNC4 and ATP8B2 were down regulated.

2.8. Analysis of the literature related to candidate genes

An ontological analysis revealed the function and the disease association of each gene, identifying genes involved in inflammation or immunity (FAM123B, ZBTB48, TNFRSF8 and IL12RB2), intercellular signaling and movement (ARGHEF16, KCNC4 and ADAM15), transcription (UTP11L and ATP8B2), cell division (PLK3, CDC7 and DCLRE1B) and keratinization (LCE1D) (14-24). After a literature review, we excluded well-known genes associated with carcinogenesis, such as PLK 3 and KCNC 4, and unidentified genes, including WDR63 and KLHDC7A. Among the remaining genes, upregulated ones were chosen as candidates. Of them, ARHGEF16 and DCLRE1B were excluded due to their unknown correlation with the tumor. Finally, IL12RB2, TNFRSF8, ADAM15 and CDC7 remained. According to previous research, these 4 genes are known to be related to inflammation, carcinogenesis or metastasis (14,16,18,19).

2.9. Quantitative reverse transcriptase polymerase chain reaction validation of gene expression

**Figure 3:**
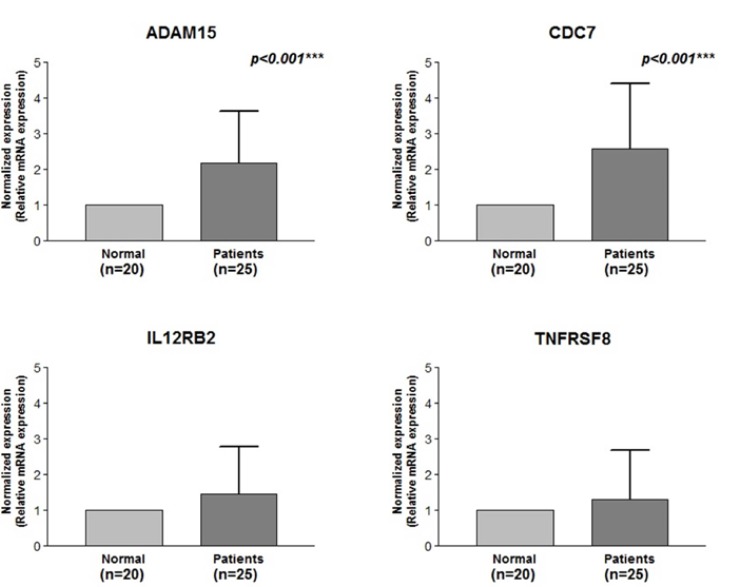
Quantitative reverse transcription polymerase chain reaction validation of microarray gene expression data from tested individuals from the Tumour Group. The quantitative reverse transcription polymerase chain reaction tests were performed with primer sets specific to ADAM15, CDC7, IL12RB2 and TNFRSF8 in 25 patients with oral squamous cell carcinoma and 20 normal matching samples. p-values (Student’s t-test) are presented. (*P < 0.05; **P < 0.01; ***P < 0.001)

Expressions of the four candidate genes (ADAM15, CDC7, IL12RB2 and TNFRSF8) were assayed by using the qRT-PCR in 28 OSCC samples to correlate microarray data with the qRT-PCR data and to explore potential biomarker expression (Fig. 3). Relative expression levels of the four genes were obtained by normalization to housekeeping gene expression levels. The qRT-PCR expression values for the four candidate genes, whose expression levels were higher in tumor tissues compared with normal tissues, were used for correlation analyses using the 2-ΔΔCt method with the Student’s t-test. In the tumor group, statistic ally-significant high mRNA expression levels of ADAM15 and CDC7 were consistently detected by using the qRT-PCR (*p*<0.001 and *p*<0.001, respectively), thus showing excellent agreement with the microarray data. A higher expression in the tumor relative to normal tissue was particularly evident for CDC7 in its increased 2-ΔΔCt value.

**Figure 4:**
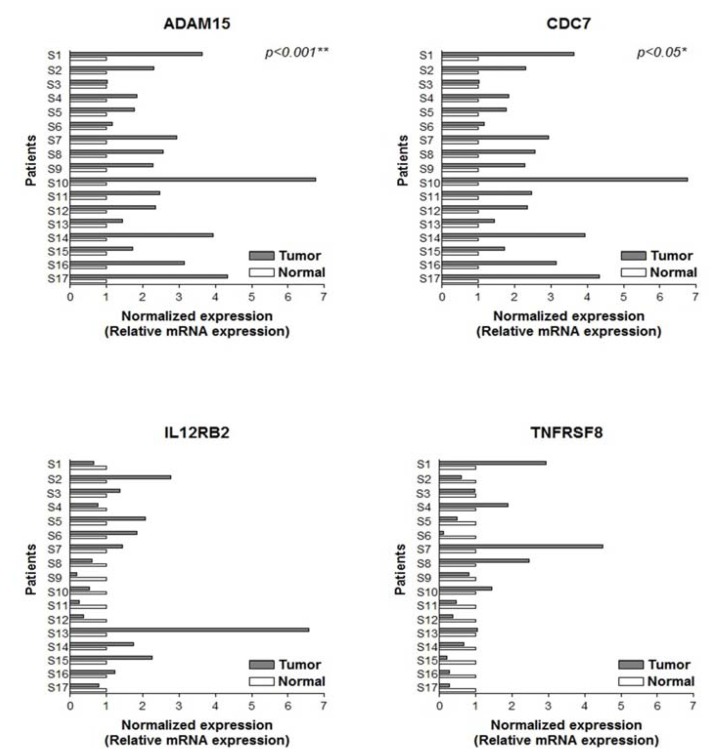
Bar chart representation of the mRNA expression of selected genes in tumour and normal patient-matched specimens (tumour–normal-paired). Normalised expression was determined by 2-ΔΔCt, by comparing threshold cycle values of ADAM15 with β-actin. Overexpression of all genes in tumour tissues is clearly demonstrated. Number of cases analysed and corresponding p-values (paired t-test) are provided. (*P < 0.05; **P < 0.01; ***P < 0.001)

We also measured the mRNA expressions of the selected genes in 17 TN-paired tissue samples by using the qRT-PCR. The normalized expression was calculated by using the 2-ΔΔCt method, as previously described, to compare normal and tumor tissues. ADAM15 expression was significantly increased in tumor tissue (*p*<0.001) by an average of 2.7-fold compared with normal tissue (Fig. 4). Consistent with these findings, upregulation of CDC7 was evident in tumor tissue (*p*< 0.05). Furthermore, the candidate genes were overexpressed regardless of variability in the degree of expression between samples in the TN-paired group.

2.10. Evaluation of the diagnostic validity of candidate genes

The relationships between the clinical parameters and the candidate genes are summarized in  Table 4. Classification of the primary site by using all four candidate genes as signatures resulted in higher accuracy (64.7%) than using the ADAM15, CDC7, IL12RB2 and TNFRSF8 genes by themselves (35.3%, 47.1%, 23.5% and 41.2%, respectively). Similar tendencies were also observed regarding tissue heterogeneity and staging. These results imply that a combination of multiple candidate genes has a discriminatory power that is superior to that achieved by using each candidate gene alone.

**Table 4:**
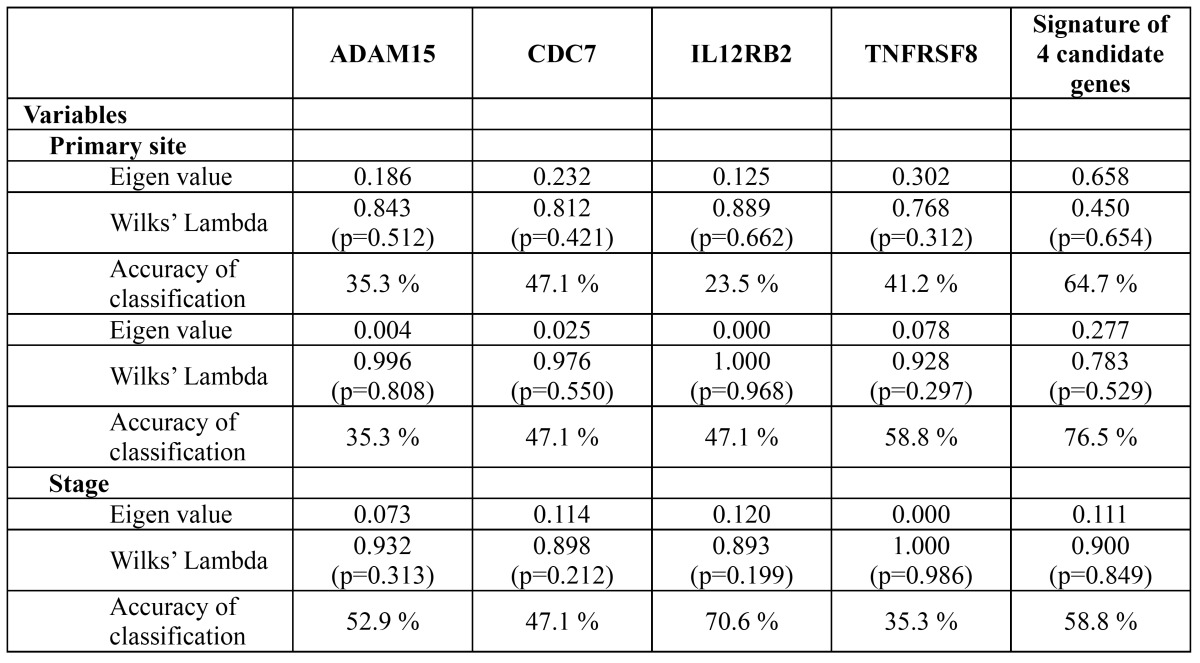
The correlation between the clinical parameters and the candidate genes determined by the discriminant analysis.

**Figure 5:**
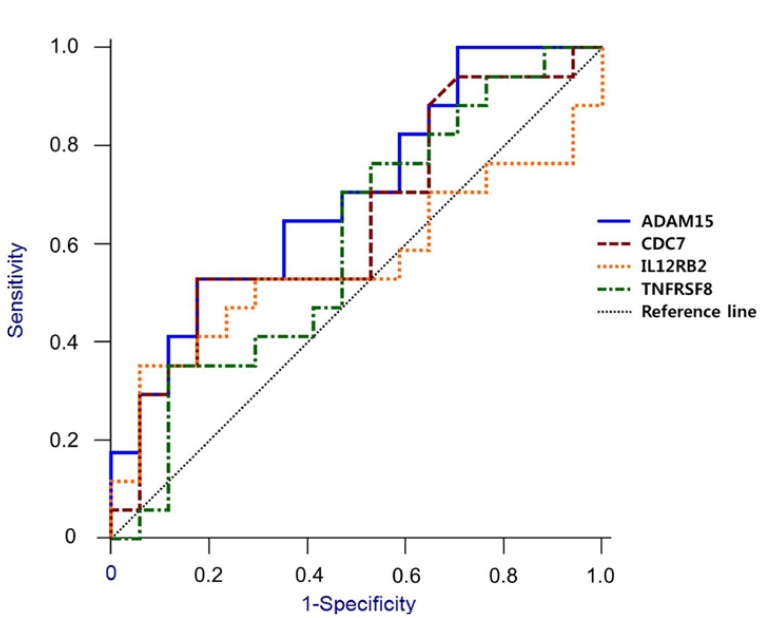
Receiver operating characteristic (ROC) curve for comparing diagnostic validity of the candidate genes. The area under the curve (AUC) of ADAM15, CDC7, IL12RB2 and TNFRSF8 were 0.699, 0.645, 0.561 and 0.602, respectively. Among them, only ADAM5 showed statistical significance (*p* < 0.05).

ROC plots are presented in fig. 5, and their diagnostic indices are shown in  Table 5. The AUCs of ADAM15, CDC7, IL12RB2 and TNFRSF8 were 0.699, 0.645, 0.561 and 0.602, respectively. ADAM15 alone, however, showed statistical significance (*p* < 0.05). Similarly, the odds ratio of ADAM15 (7.271; *p* = 0.029) and CDC7 (26.794; *p* = 0.046) were statistically significant.

**Table 5:**
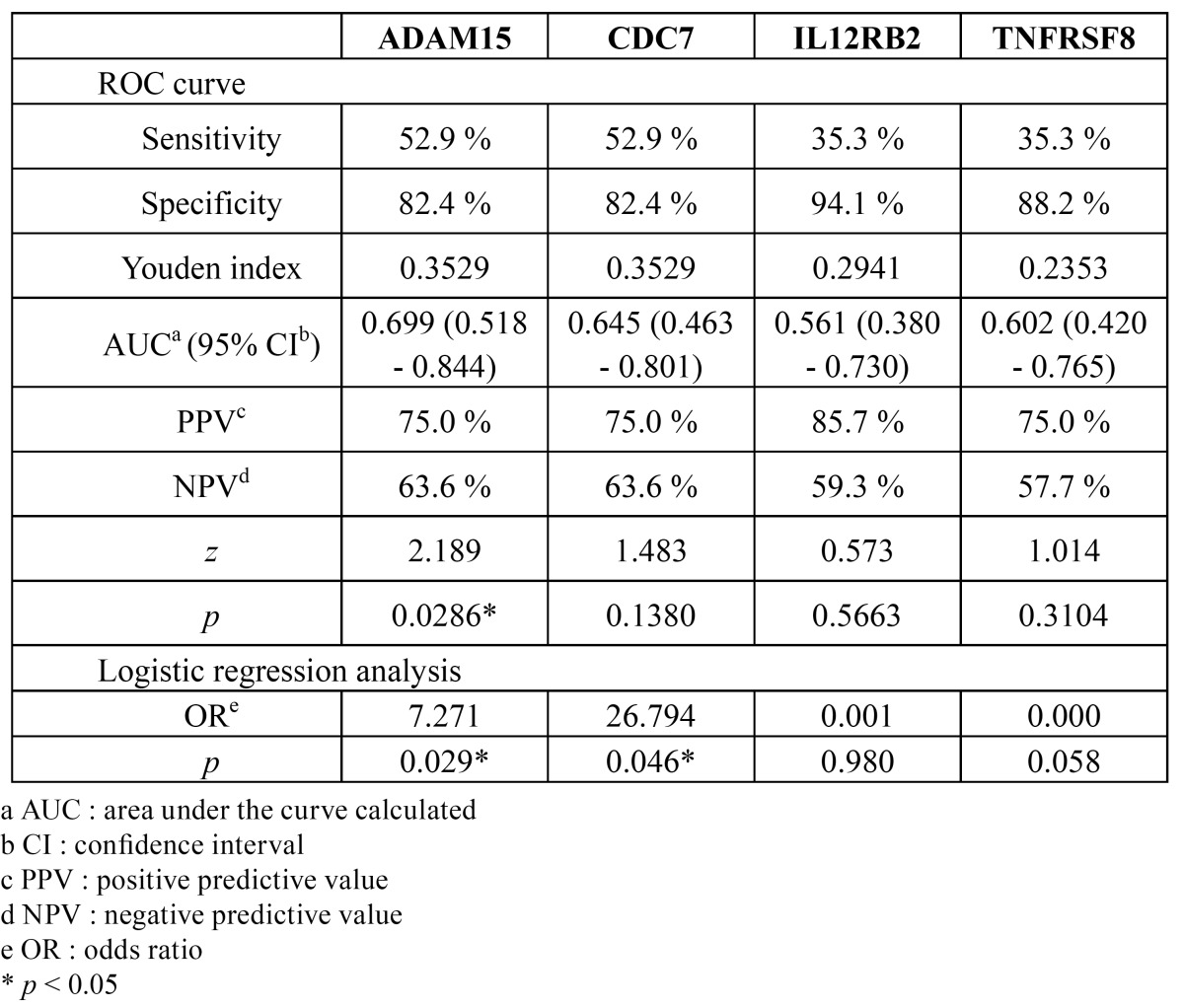
The diagnostic validity of the candidate genes for OSCC determined by ROC curves and logistic regression analysis.

## Discussion

The five-year survival rate of patients with an OSCC has remained about 50% for several decades. This is one of the worst prognoses of all cancers and is directly related to the advanced stage at diagnosis (25). Local recurrence and distant metastasis may develop despite an apparently complete excision and histopathologically tumor-free margins, supporting the theory of field cancerization (26). Novel biomarkers could allow a more sensitive and accurate surgical margin to be determined. However, genome research has been hindered by individual and ethnic differences (27).

Although the sample size of the Microarray Study group (n = 5) seems too small, we tried to ensure accurate results in three ways: (1) several statistical analyses (the permutation test, the LPE and the SAM), which can identify significant DEGs with a small number of microarrays, were applied (10), (2) genes carefully selected by using these statistical tools were validated via the qRT-PCR with sufficient samples, and (3) as shown in  Table 1, patients’ clinicopathological characteristics were evenly distributed in both the Microarray Study and the qRT-PCR Validation groups. In spite of a general consensus that smoking history is a major contributor to carcinogenesis (28), gene expression between smoking and nonsmoking subjects did not differ in this study. Further, Méndez *et al*. observed no statistic ally-significant differences in gene expression between patients with different histopathologic stages when using oligonucleotide arrays (29). Thus, the effect of smoking history and histopathological type on our results can be expected to be minimal.

To date, many studies have attempted to identify novel biomarkers for an OSCC (3,6,30). However, they were not generally accepted for the following reasons: First, candidate biomarkers yielded by the microarray analysis were usually identified based on fold changes (30), but these can often be misleading, as different error variances exist under different biological conditions and in different microarray expression intensity ranges (10). Second, most previous studies mainly focused on well-known genes, such as matrix metalloproteinases (MMPs), p53 and VEGF (6,8). Such an approach could result in a bias against unknown genes. In this study, we tried to overcome possible bias by using a microarray analysis (a relatively unbiased high-throughput method) and to reduce the statistical error by using four kinds of biostatistics verification.

From our results, 15 genes were initially screened ( Table 3). As previously mentioned, genes with unknown functions were excluded, and tumor-related ones (ARHGEF16, DCLRE1B, TNFRSF8, PLK3, IL12RB2, CDC7, KCNC4 and ADAM15) were selected. After that, upregulated genes (ARHGEF16, DCLRE1B, TNFRSF8, IL12RB2, CDC7 and ADAM15) were selected according to hypothesis that upregulated biomarkers are more valuable because their overexpression will increase the sensitivity of diagnosis (31). Among them, 4 genes (ADAM15, CDC7, IL12RB2 and TNFRSF8) were selected for further evaluation because of their involvement in malignancy.

ADAM15 is the only member of the disintegrin and metalloproteinase (ADAM) family and is involved in T-cell-mediated immune responses and pathologic neovascularization (32). It is upregulated in breast, stomach, lung, pancreas, and prostate cancer and in metastatic progression (33). Conversely, its tumor suppression in melanomas and colon carcinomas was also proposed (34,35). This inconsistency might result from the complexity of the ADAM15-related signal transduction pathways. In this study, ADAM15 was significantly upregulated in patients with an OSCC.

CDC7, a serine threonine kinase, is a core component of the initiation machinery of DNA replication, and its inhibition in cancer cells induces p53-independent apoptosis (36). Clinically, in many kinds of cancers, CDC7 upregulation is related to tumor anaplasia, aneuploidy, advanced disease stage and lower relapse-free survival (37-39). These results agreed with our data, which showed over expression of CDC7 in patients with an OSCC.

IL12RB2 encodes a type-1 transmembrane protein, a sub unit of the Interleukin 12 (IL-12) receptor complex. IL12RB2 behaves as a tumor suppressor in human chronic B-cell malignancies, and its silencing is an early event in B-cell malignant transformation (27,40). Prolonged disease-free survival has also been reported to have been found in colorectal cancer patients with higher expressions of IL12RB2 (41). However, the correlation between IL12RB2 expression and OSCC development is unclear. Considering that interleukin 6 (IL-6) and IL-12 functionally antagonize each other and that IL-6-induced inflammation promotes carcinogenesis in the oral cavity (42,43), IL12RB2 down regulation might be involved with an OSCC. However, despite over expression of IL12RB2 in patients with an OSCC in the Microarray Analysis group, no significant differences of IL12RB2 expression were found in our qRT-PCR data.

TNFRSF8 is known to function in apoptosis by activating the caspase pathway (44) and protecting the body against auto immunity. It is a conventional biomarker of hematologic malignancies (45), but its relationship with solid tumors is unclear. Only one study reported that its over expression might be associated with improved prognosis for patients with a skull base chordoma (46). Though TNFRSF8 in patients with an OSCC was upregulated in the microarray analysis, qRT-PCR validation showed no significant differences. Hence, further investigation is needed.

Among the four candidates, ADAM15, IL12RB2 and TNFRSF8 are known to be related to inflammation. Given that the oral cavity is vulnerable to frequent inflammatory stress and that this environment can affect cancer cells, our results support that inflammation might be correlated with OSCC development, as reported in some recent studies (6,43).

 Table 4 presents the correlation between the candidate genes and the clinical parameters (primary site, tissue heterogeneity and stage). Although all p values were higher than 0.05, the eigenvalues and the accuracies of the signatures of the four genes for each parameter were generally lower than those of individual genes according to the Wilk’s lambda distribution analysis. This result indicates that the classifying power of the four genes combined was superior to that of each gene alone.

As presented in  Table 5, the sensitivity, specificity and predictive values were calculated at the cut-off points according to the Youden index. At the cutoff points, the value of [sensitivity + specificity - 1] is a maximum (47). The Youden index is widely used to determine the diagnostic threshold, and it is regarded as the most appropriate method of combining biomarkers (48). In terms of the AUC, ADAM15 showed higher clinical efficacy (*p* < 0.05) compared to the other candidate genes. The odds ratios of ADAM15 and CDC7 also showed statistically-significant correlations to an OSCC (*p* < 0.05).

In conclusion, four genes (TNFRSF8, IL12RB2, CDC7 and ADAM15) were presented as candidates for OSCC biomarkers by using a microarray analysis and a qRT-PCR validation with biostatistical verification. Further investigations are needed in order to construct more accurate diagnostic models. To our knowledge, this is the first report on OSCC biomarkers in Korean patients.
